# Virulence of atypical *Toxoplasma gondii* strains isolated in French Guiana in a murine model

**DOI:** 10.1051/parasite/2019048

**Published:** 2019-09-24

**Authors:** Stéphane Simon, Benoit de Thoisy, Aurélien Mercier, Mathieu Nacher, Magalie Demar

**Affiliations:** 1 Equipe EA3593 – Ecosystèmes Amazoniens et Pathologie Tropicale, Université de la Guyane 97300 Cayenne French Guiana; 2 Laboratoire de Parasitologie – Mycologie, Cayenne General Hospital 97300 Cayenne French Guiana; 3 Laboratoire des Interactions Virus-Hôtes, Institut Pasteur de la Guyane 97300 Cayenne French Guiana; 4 INSERM UMR_S 1094, Neuroépidémiologie Tropicale, Laboratoire de Parasitologie-Mycologie, Faculté de Médecine, Université de Limoges Limoges 87025 France; 5 Centre National de Référence Toxoplasmose/Toxoplasma Biological Ressource Center, CHU Limoges 87042 Limoges France; 6 Centre d’Investigation Clinique Epidémiologie Clinique Antilles Guyane CIC CIE 1424, Cayenne General Hospital 97300 Cayenne French Guiana

**Keywords:** Toxoplasmosis, Amazonian, French Guiana, Virulence, Mice

## Abstract

*Background*. *Toxoplasma gondii* is an obligate intracellular protozoan parasite of warm-blooded vertebrates. Most infections in immunocompetent patients are asymptomatic. However, since 2000s, strains with particular genetic profiles that differ from the known clonal type (type I, II, III), have been described. In French Guiana, these strains are highly pathogenic in immunocompetent patients. They have defined a new clinical entity called Amazonian Toxoplasmosis. The present study aims to further improve our knowledge on the pathogenicity of these Amazonian *T. gondii* strains in comparison with three reference strains using Swiss strain mice. With these data, we tried to establish a predictive virulence score to classify these strains, but also to correlate this virulence with the severity of the disease in infected patients. *Results*. All the virulence indicators revealed that the Amazonian strains isolated in French Guiana presented a high virulence profile, but lower than the highly virulent type I reference RH strain. The findings reveal differences in virulence between human and animal strains, but also between anthropized and wild strains. *Conclusion*. In addition to being a clinically relevant animal model of Amazonian Toxoplasmosis, this model could also provide a solid experimental basis for future studies aiming to investigate the underlying mechanisms of Amazonian Toxoplasmosis disease.

## Abstract (fr)

*Contexte. Toxoplasma gondii* est un parasite protozoaire intracellulaire obligatoire des vertébrés à sang chaud. La plupart des infections chez les patients immunocompétents sont asymptomatiques. Cependant, depuis les années 2000, des souches avec des profils génétiques particuliers qui diffèrent du type clonal connu (type I, II, III) ont été décrites. En Guyane française, ces souches sont hautement pathogènes chez les patients immunocompétents. Elles ont défini une nouvelle entité clinique appelée Toxoplasmose Amazonienne. La présente étude vise à approfondir nos connaissances sur le pouvoir pathogène de ces souches amazoniennes de *T. gondii* par rapport à 3 souches de référence en utilisant des souris de souche Swiss. Avec ces données, nous avons tenté d’établir un score de virulence prédictif pour classer ces souches mais également de corréler cette virulence avec la gravité de la maladie chez les patients infectés. *Résultats*. Tous les indicateurs de virulence ont révélé que les souches amazoniennes isolées en Guyane française présentaient une virulence élevée mais plus faible que la souche de référence RH très virulente de type I. Les résultats ont mis en évidence les différences de virulence entre les souches humaines et animales mais aussi entre les souches anthropisées et les souches sauvages. *Conclusion*. En plus d’être un modèle animal cliniquement pertinent de la toxoplasmose amazonienne, ce modèle pourrait également fournir une base expérimentale solide à de futurs travaux qui chercheront à approfondir les mécanismes sous-jacents de la toxoplasmose amazonienne.

## Introduction

Discovered since 1908 [38, 51], *Toxoplasma gondii* is a parasite of warm-blooded vertebrates, including mammals, birds and humans [17]. This obligate intracellular protozoan is a cosmopolitan pathogen that is estimated to infect nearly one-third of the adult human population [24]. Humans are contaminated either by consumption of raw or undercooked meat containing tissue cysts or by accidental consumption of oocyst-contaminated vegetables or drinking contaminated water [23, 25, 29]. Until the last two decades, infection with *T. gondii* was usually considered to cause no illness, but only some mild clinical signs in immunocompetent individuals, specifically transient lymphadenopathy and mild flu-like presentations. Severe outcomes such as encephalitis, pneumonia, myocarditis, or disseminated infections were uncommon. It is well-known that infections acquired during pregnancy can result in mild to serious congenital defects in the fetus, and potentially evolve toward ocular infections [34, 40]. In immunocompromized individuals, such as transplant recipients and patients living with HIV, toxoplasmosis can result in severe consequences, including encephalitis, chorioretinitis, or myocarditis, which can potentially be fatal [4]. *Toxoplasma gondii* strains isolated in North America and Europe have been grouped into three major clonal lineages as types I, II, and III, based on six genetic markers [8, 26]. Several studies on experimental *T. gondii* infections have reported a correspondence between virulence in mice and genotype: type I strains such as RH are the most virulent with a 100% lethal dose (LD_100_) of one parasite [47] less than 10 days after inoculation. In contrast, type II strains such as PRU have intermediate virulence (LD_50_ > 10^3^), whereas type III strains such as VEG are almost avirulent (LD_50_ > 10^5^) [49]. There may also be differences in the virulence of the three strains in humans [4, 8, 26]. The development of genetic analyses by RFLP-PCR [52] or microsatellites [5] showed more complex polymorphism for several strains isolated from Africa [6, 19, 22, 37], Asia [28, 32, 42] and South America [45, 53]. However, despite this complex polymorphism, some of these strains isolated from humans and animals can be grouped into haplogroups corresponding to clonal lineages across these continents [30, 31, 33, 36]. In French Guiana, an Amazonian tropical area, emerging strains exhibit high genetic diversity with strains belonging to different haplogroups (HG). It revealed that anthropized strains were closely grouped with HG3, while wild strains were grouped with HG5 or HG10 [31]. These strains can be responsible for a new clinical entity called Amazonian Toxoplasmosis. This is a severe form of systemic acquired toxoplasmosis among immunocompetent adults, with greater potential for death [2, 3, 9, 10, 13–16, 37, 46].

The present study aimed to improve our knowledge of the pathogenicity of these Amazonian *T. gondii* strains in comparison with the three reference strains using a murine model, and calculated mortality-linked parameters.

## Materials and methods

### Ethics statement

All experiments carried out in mice in this study were in agreement with the recommendations of European Directive No. 86/609/EEC and French Decree No. 2013-118. The experimental protocols were approved by the Veterinary Services Department and by the Ethics Committee of the Institut Pasteur under the reference C2EA 89 – CETEA Institut Pasteur.

### Experimental mice and parasites

Specific pathogen-free (SFP) Swiss CD1 mice were used (Charles River Laboratory, Lyon, France), and they were bred at the animal facility of Institut Pasteur de la Guyane.

In addition to the reference strains RH (type I), PRU (type II) and VEG (type III), 10 *T. gondii* strains isolated in French Guiana and genotyped by the microsatellite method (Table 1) were provided by the Biological Resource Center of Limoges (France). Five of them were isolated from patients and the other five stains were isolated from wild or domestic animals [3, 15, 37]. The tachyzoites were cultured and harvested by serial passages on monolayers of Human Foreskin Fibroblast cells (HFF-1, ATCC^®^ number: SCRC-1041^™^) in Roswell Park Memorial Institute medium (RPMI) 1640 with L-glutamine (PAN Biotech GmbH, Aidenbach, Germany) supplemented with 10% heat-inactivated fetal bovine serum (FBS) (PAN Biotech GmbH, Aidenbach, Germany), penicillin (100 U/mL) and streptomycin (100 μg/mL) (PAN Biotech GmbH, Aidenbach, Germany) at 37 °C in a humid 5% CO_2_ atmosphere. HFF-1 monolayers were infected by 10^5^ tachyzoites and after 1 week, all cells were lyzed. The tachyzoites were scraped and counted into a Kova cell chamber. The tachyzoites were diluted to the appropriate concentration in sterile isotonic saline (PBS). For each strain, different doses of tachyzoites ranging from 5, 5 × 10^1^, 5 × 10^2^, 5 × 10^3^ and 5 × 10^4^ were respectively inoculated intraperitoneally into groups of 10 mice (Group 1, Group 2, Group 3, Group 4, and Group 5). The parasite load of the inoculum was checked using *Toxoplasma* real-time PCR (Bio-Evolution, Bry-sur-Marne, France), according to the manufacturer’s recommendations.

**Table 1:** Characteristics of the *Toxoplasma gondii* strains used in this study.

Isolate	BRC code number	MS genotype	References	Origin	Host	Patient symptoms
RH	ND	Type I	[48]	North America	Human	
PRU	TgH00001	Type II	[30]	France	Human	
VEG	TgH00005	Type III	[48]	North America	Human	
GUY-WAY-2007	TgH18028	Amazonian	[37]	Maripasoula area*	Human	Fever, persistent bronchitis (severity: low)
						Hospitalization
						Favorable course with treatment
GUY-GRO-2011	TgH18049	Amazonian	N.C.	Maripasoula area*	Human	Fever, hepatitis (severity: intermediate)
						Hospitalization
						Favorable course with treatment
GUY-MEL-2003	TgH18007	Amazonian	[37]	Mana river*	Human	Fever, pneumopathy, heart damage (severity: intermediate) Hospitalization
						Death from heart damage, 2 years after treatment
GUY-AKO-2004	TgH18009	Amazonian	[37]	Apatou area*	Human	Fever, heart failure, anasarca (severity: high)
						Intensive care unit
						Favorable course with treatment
GUY-TOJ-2006	TgH18021	Amazonian	[37]	Apatou area*	Human	Fever, general failure (severity: very high)
						Intensive care unit
						Death despite treatment
GUY-CAN-FAM-0001	TgA18002	Caribbean1	[37]	Macouria*	Dog (*Canis familiaris*)	
GUY-CAN-FAM-0002	TgA18004	Caribbean2	[37]	Macouria*	Dog (*Canis familiaris*)	
GUY-CAN-FAM-0007	TgA18006	Amazonian	[37]	Roura*	Dog (*Canis familiaris*)	
GUY-JAG-2004	TgA18001	Amazonian	[37]	Belizon track*	Jaguar (*Panthera onca*)	
GUY-GAL-VIT-0001	TgA18005	Single isolate	[37]	Matoury*	Grison (*Galictis vittata*)	

Human Guyanese strains are classified by the severity of clinical signs observed. Animal strains are classified by the domestic or wild biotope.

N.C.: Not communicated.

* Sites localized in French Guiana.

### Dose effect and mouse virulence tests

The virulence of *T. gondii* strains was determined by monitoring clinical signs and cumulative mortality after peritoneal injection. We maintained 10 mice as uninfected controls (Group 0) by injecting sterile isotonic saline (PBS). The mice were clinically observed daily for 31 days. During the observation period, dead mice were counted. At the end of the observation period, live mice were counted and euthanized. Toxoplasma infection was controlled using *Toxoplasma* real-time PCR (Bio-Evolution) on the heart samples of the dead or euthanized mice

### Statistical analysis

#### Survival curve

Kaplan-Meier [41] plots were plotted, and the Log-rank test was used to compare survival of mice and the mortality data such as the mortality rate (MR) of the experimental groups. *p* values *≤*0.05 were considered significant.

#### Dose effect curve

The dose effect analysis was based on a probit regression to model the impact of tachyzoite doses on the survival of a group of mice. The analysis made it possible to determine LD_50_ and LD_99_ which are respectively the required number of parasites to kill 50% or 99% of infected mice. Statistical analyses and graphics were performed using XLSTAT-Biomed [1].

### Predictive score of virulence

Virulence is generally defined as the intensity of an infectious microorganism’s pathogenicity for its host. To better define and objectively quantify *T. gondii* virulence in mice, we determined a predictive virulence score for each strain. For this, we used several parameters such as injected dose, infected population, median number of days of survival after infection, and mortality rate, and defined the virulence score as:

(1) $$\mathrm{Virulence}\;\mathrm{score}\;(\mathrm{VS})\;=\mathrm{Average}\left(\sum \left(\left(\frac{1}{\log(n_{\mathrm{tac}hy})}\times \frac{1}{\mathrm{median}\;\mathrm{survival}}\right)\times \left(\frac{n_{\mathrm{deat}h}\times 100}{n_{\mathrm{injected}}}\right)\right)\right)$$

*n*_tachy_: number of injected tachyzoites,

*median survival*: median of survival days per the number of injected tachyzoites,

*n*_death_: number of mice that died per the injected dose,

*n*_injected_: number of injected mice.

This score was used to objectively compare the different *T. gondii* strains with each other, especially strains of unknown virulence versus reference strains.

## Results

The appearance of the disease in mice is characterized by clinical signs (respiratory distress, ruffled hair, asthenia, sluggishness). Death occurs, in general, between 3 and 5 days after the appearance of the first signs. Autopsy of the mice showed significant damage to the heart, lungs and brain (data not shown). These pathophysiological features are similar to those seen in the most severely infected patients.

The presence of *Toxoplasma gondii* was verified by qPCR on heart samples of all the mice (either dead during the experiment, or euthanized after the 31 days of testing). All samples were positive confirming the presence of *T. gondii* in all mice.

### Survival curves in infected mice

**Figure 1:**
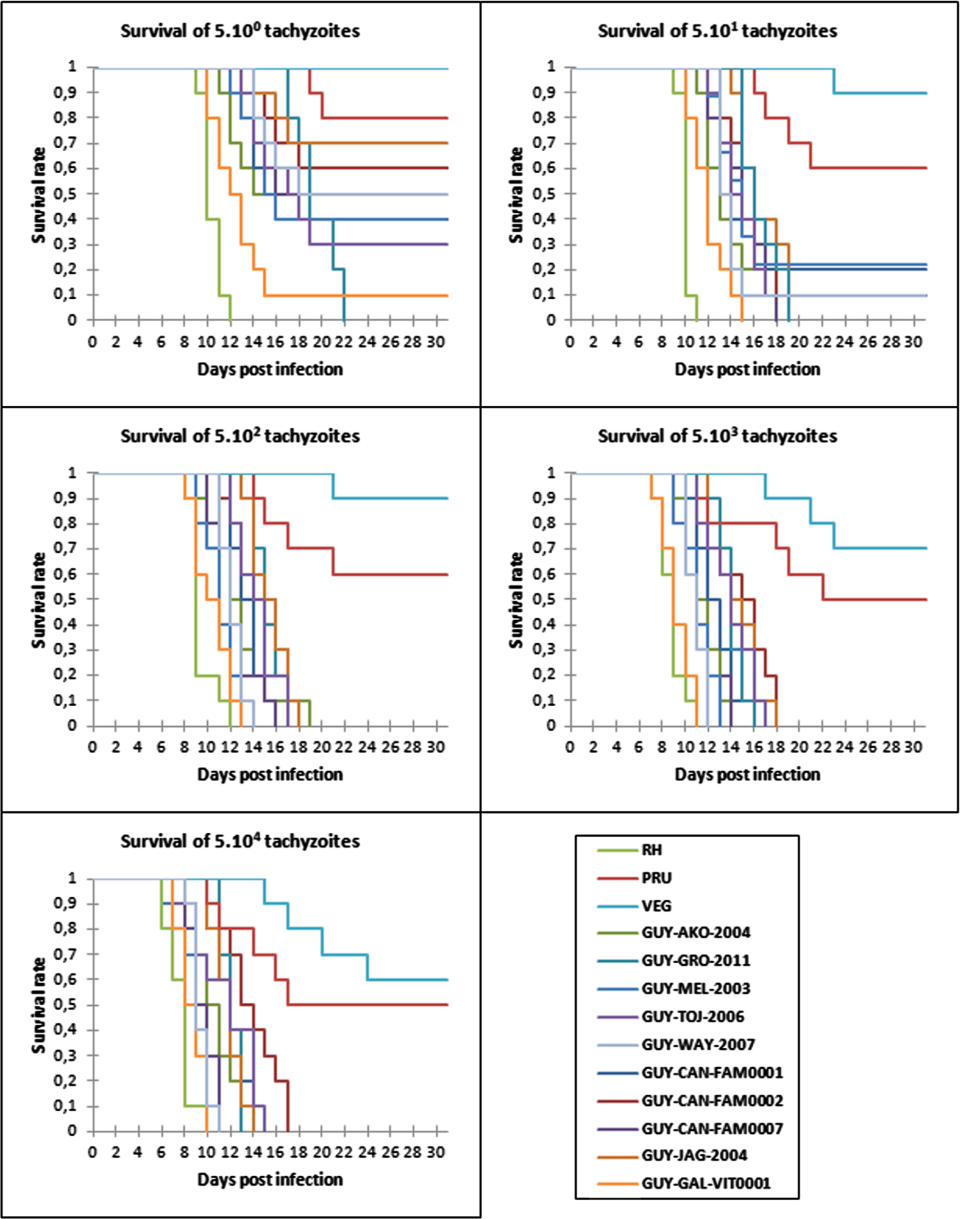
Virulence in mice of atypical *Toxoplasma gondii* strains isolated in French Guiana compared to reference strains (type I, II and III). Ten mice/groups were inoculated intraperitoneally with different doses of tachyzoites for each strain, and the viability of mice was monitored daily. Survival was followed up for 31 days after injection and data were plotted as Kaplan-Meier curves (statistical significance of survival curves between RH and others strains, *p ≤* 0.001).

The differences of mice survival depending on the infecting dose injected are shown in Figure 1. Unsurprisingly, we confirmed that the greater the injected dose, the higher the number of deaths observed.

When 5 tachyzoites were injected, the RH and GUY-GRO-2011 strains killed the entire group of mice in 12 days and 22 days, respectively. For the other Guianese strains, the MR was less than or equal to 70% after 31 days except the GUY-GAL-VIT0001 strain whose MR is 90% at 15 days. It was 20% and 0% after 31 days for the PRU (type II) and VEG (type III) strains, respectively.

When 50 tachyzoites were injected, the GUY-CAN-FAM0007 and GUY-GAL-VIT0001 strains killed the entire group of mice in 18 days and 15 days, respectively. For 5 strains such as GUY-AKO-2004, GUY-TOJ-2006, GUY-WAY-2007, GUY-CAN-FAM0002 and GUY-JAG-2004, the MR was 90%. It was 40% and 10% for PRU and VEG strains, respectively.

When 500 tachyzoites were injected, all the Guianese strains had a 100% MR between 13 and 19 days, whereas the PRU and VEG strains had mortality rates of 40% and 30%, respectively.

The higher the injected dose of tachyzoites, the shorter the time to death (Table 2). The median survival time of mice injected with 5 tachyzoites (minimum doses) for the RH strain was 10.4 days (with an MR of 100%), 19.5 days for PRU (with an MR of 20%), and there was no mortality for VEG. The median survival time with 5 × 10^4^ tachyzoites (maximum dose) for the RH strain was 7.6 days (with an MR of 100%), 14.5 days for PRU (with an MR of 50%), and 19 days for VEG (with an MR of 40%). For Guianese strains, the median survival time ranged between 12.1 (GUY-GAL-VIT0001) and 19.5 (GUY-GRO-2011) days for a dose of 5 tachyzoites and between 8.6 (GUY-GAL-VIT0001) and 13.8 (GUY-CAN-FAM0002) days for an inoculum of 5 × 10^4^ tachyzoites injected (Table 2).

**Table 2:** Median survival days (±*SD*) of mice inoculated with different doses of parasites.

		*T. gondii* strains												
		RH	PRU	VEG	GUY-AKO-2004	GUY-GRO-2011	GUY-MEL-2003	GUY-TOJ-2006	GUY-WAY-2007	GUY-CAN-FAM 0001	GUY-CAN-FAM 0002	GUY-CAN-FAM0007	GUY-JAG-2004	GUY-GAL-VIT0001
Doses of parasites	5 × 10^0^	10.4 ± 0.5	19.5 ± 1.0	Undefined	13.3 ± 2.0	19.5 ± 1.9	14.2 ± 1.2	15.7 ± 1.8	15.4 ± 1.5	13.3 ± 0.9	15.8 ± 1.7	15.3 ± 1.2	15.7 ± 1.5	12.1 ± 1.2
	5 × 10^1^	10.0 ± 0.3	18.3 ± 2.2	23	13.2 ± 1.2	16.5 ± 1.0	13.8 ± 1.0	14.4 ± 1.1	13.6 ± 0.5	14.0 ± 1.2	15.6 ± 1.3	14.9 ± 1.3	16.2 ± 1.3	12.0 ± 1.0
	5 × 10^2^	9.4 ± 0.7	16.8 ± 3.0	21	12.8 ± 1.7	15.3 ± 0.7	12.0 ± 0.7	14.3 ± 1.1	12.2 ± 1.0	13.4 ± 0.9	14.7 ± 1.5	12.4 ± 1.2	15.4 ± 1.0	10.4 ± 1.0
	5 × 10^3^	8.8 ± 0.7	16.2 ± 3.9	20.3 ± 3.5	11.5 ± 1.0	14.0 ± 0.7	11.1 ± 0.9	13.9 ± 1.3	10.9 ± 0.5	12.6 ± 1.8	14.8 ± 1.6	11.5 ± 0.9	14.4 ± 1.3	9.2 ± 0.8
	5 × 10^4^	7.6 ± 0.7	14.5 ± 2.8	19.0 ± 3.8	10.3 ± 1.4	12.1 ± 0.5	8.9 ± 0.9	11.7 ± 1.7	9.4 ± 0.5	11.9 ± 0.9	13.8 ± 1.5	9.6 ± 0.7	11.8 ± 0.8	8.6 ± 0.7

### Determination of LD_50_ and LD_99_

**Figure 2:**
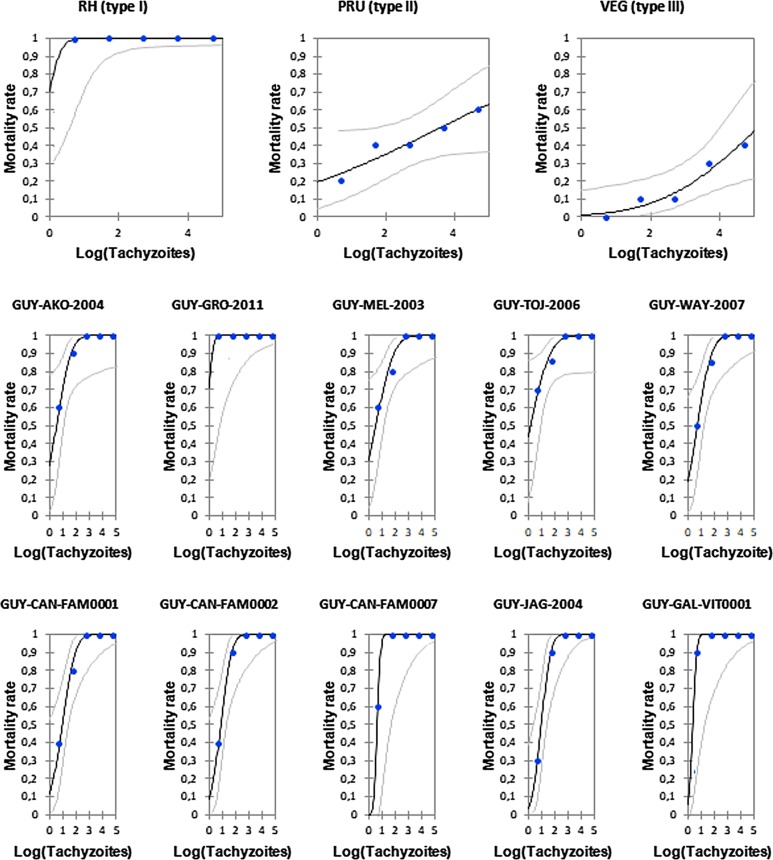
Determination of LD_50_ and LD_99_ for the 10 atypical strains of *Toxoplasma gondii* compared to reference strains (type I, II and III). Dose effect curves using a Probit model (black). Confidence interval (95%) curves are also shown (grey). On top of the figure, the tree reference strains (I, II and III) are shown for comparison.

The mortality rate (MR) of the mice after 31 days post infection according to the strain is shown in Figure 2. The profile of the three reference strains is very specific. The RH strain showed a highly virulent profile with 100% mortality for a very small amount of injected tachyzoites. The PRU strain had a less aggressive profile with an MR between 20% and 50%, and the VEG strain was almost as virulent because the mortality rate varied from 0% to 40% for a number of injected tachyzoites ranging from 5 to 5 × 10^4^. We highlighted that, when compared with the reference strains, the Guianese strains had profiles close to the RH strains.

We used the line equation to calculate LD_50_ and LD_99_ which are respectively the required amount of parasites to kill 50% or 99% of infected mice (Table 3). The reference strains RH, PRU and VEG had an LD_50_ of 1, 3933 and 126,373 tachyzoites, respectively and an LD_99_ of 4, 2 × 10^13^ and 1.5 × 10^10^ tachyzoites, respectively. French Guianese strains had an LD_50_ value ranging between 1 and 10 tachyzoites, and an LD_99_ value between 7 and 1018 tachyzoites. These values confirm the virulent profile of the Guianese strains.

**Table 3:** Determination of LD_50_ and LD_99_.

	*T. gondii* strains												
	RH	PRU	VEG	GUY-AKO-2004	GUY-GRO-2011	GUY-MEL-2003	GUY-TOJ-2006	GUY-WAY-2007	GUY-CAN-FAM 0001	GUY-CAN-FAM 0002	GUY-CAN-FAM0007	GUY-JAG-2004	GUY-GAL-VIT0001
DL_50_	1	3933	126,273	3	1	3	2	5	8	7	4	10	2
DL_99_	4	2E + 13	1.5E + 10	327	7	1018	745	465	561	217	12	182	9

### Virulence score in mice

**Figure 3:**
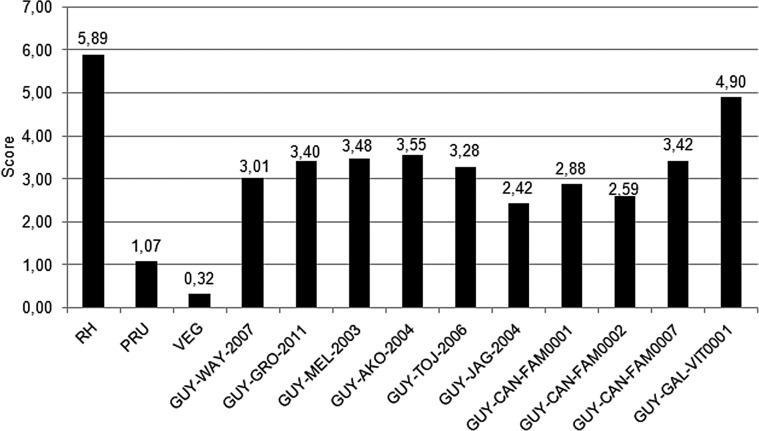
Scoring of virulence of *Toxoplasma gondii* strains isolated in French Guiana compared to reference strains type I, II and III. The first three strains are the reference strains, the next five strains are isolated from patients (with an increase in the severity of the symptoms), and the last five strains are isolated from animals (three from domestic fauna and two from wildlife).

We first determined the levels of virulence in murine models for the reference strains (Fig. 3). The highly virulent RH strain corresponded to a high score of 5.89. The intermediate virulent PRU strain had a score of 1.07, and the non-virulent strain had a low score of 0.32.

We then compared the virulence scores with the severity of the symptoms caused in infected patients. Patients were classified with increasing severity (Table 1), thus virulence scores should also increase. There was an increase in the score for the first four patients (GUY-WAY-2007, GUY-GRO-2011, GUY-MEL-2003 and GUY-AKO-2004) who had a virulence score of 3.01, 3.40, 3.48 and 3.55, respectively. However, the last patient had the most severe symptoms (death), but his score was only 3.28. We hypothesized that scores would increase with increasing symptom severity in patients. This was confirmed in the first four patients, but this increase was very low compared to the symptoms in the different patients. The difference in score between a patient with persistent bronchitis and a patient in an intensive care unit was only 0.54.

The scores determined for the strains isolated from animals were more heterogeneous ranging from 2.42 for the GUY-JAG-2004 strain to 4.9 for the GUY-GAL-VIT0001 strain.

The Guianese strains ranged between the highly virulent RH strain and the intermediate virulent PRU strain. This seems to indicate rather high virulence especially for the GUY-GAL-VIT0001 strain, which showed a very high score. However, although its mortality rate was not the highest, its score was high because the death of the mice occurred very quickly after the injection.

## Discussion

To our knowledge, the present experimental results on *Toxoplasma* virulence in mice are original. Most articles explore the virulence in mice from very specific approaches. Some experiments are much more focused on analyzing the correlation between clinical data and biological markers of acute, chronic or secondary infections involving European or North-American strains [27], while others attempt to demonstrate the basic patterns of *T. gondii* pathophysiological mechanisms using transgenic mice [11, 19, 44] or other advanced technologies, such as bioluminescence imaging [7]. Some general reviews reported data from experimental assays and overviewed hypotheses underlying toxoplasma virulence based on general comparisons between different genotypic strains including the clonal reference ones [18, 21, 35, 50]. Recently, Saraf et al. [43], proposed a standardized methodology for future studies in order to enable more efficient and effective analysis of genetics and virulence patterns for *T. gondii*.

Our study, despite being performed before the publication of this article, complied with its instructions except for: (i) the determination of seroconversion by modified agglutination test (MAT) as we used *T. gondii* qPCR to confirm the infection, (ii) the number of mice injected per group, 10 instead of 5, in order to improve the performance and robustness of the study, and (iii) the studied parameters, such as the determination of the DL_100_ and DL_99_, and calculated the cumulative mortality rate using another approach. In fact, we concentrated on determining a virulence score by considering several relevant parameters such as the survival time, the amount of injected tachyzoites, and the cumulative MR.

This parameter seemed to be an objective and useful tool to evaluate virulence in mice. It matched with the other results of this study and was validated by the good correspondence of its values with clinical classes (virulent, intermediate and non-virulent) for the reference strains. It could thus be used to compare the strains among one another, especially strains isolated from diverse geographical areas, and enabled us to correlate the virulence of these strains with the clinical features in humans, when there are human contaminations. We emphasize that this experiment obviously cannot be extrapolated to other experimental rodent species: susceptibility to *T. gondii* infection varies among different hosts [12, 17] and laboratory mice, generally sensitive, are often used as the preferred animal model to determine parasite virulence.

The RH strain (type I) had a 100% mortality rate with five injected tachyzoites. The PRU (type II) and VEG (type III) strains did not have a 100% mortality rate. However, all these Guianese strains had a 100% mortality rate with only 500 tachyzoites injected. This suggests that the atypical *T. gondii* strains circulating in French Guiana have virulence factors comparable to those established by the type I strain compared to those of type II or type III. This fitted with the results of the virulence score. Regarding this parameter, we should highlight the differences between strains isolated from humans and animals, and even within the strains isolated from animals. It is unclear whether this should be explained by the geographic origin of the strains or by the type of intermediate or definitive hosts clearly, recent studies revealed that in French Guiana, there are two *T. gondii* environmental populations, the wild population with very high genetic diversity, and the anthropized population presenting lower genetic diversity [37].

Thus, in this study, the strains isolated from humans (Amazonian type) showed elevated high virulence scores with a median of 3.34, but lower than that of the RH strain (5.89). According to patient surveys and their reported risk factors [9, 16], these strains were related to wild environments. Except for two patients who developed mild pneumopathy (GUY-WAY-2007, GUY-MEL-2003), the others presented severe infections with organ failure (heart, kidneys, liver, lungs). Patients were classified by the degree of severity of symptoms (Table 1). However, the classification of patients by severity was difficult because other factors than the infecting strain can influence the intensity of human disease (host genetic factors, inocula, etc.). The low variation of the virulence score between the patient with pneumopathy (GUY-WAY-2007) and the patient with cardiac involvement (GUY-AKO-2004) did not reveal an obvious correlation between the degree of severity and the virulence score. In addition, the last patient had the most serious symptoms but his score was one of the lowest because this patient was already weakened during the contamination, unlike the other four.

However, these scores are higher than for the PRU (type II) strain, indicating fairly strong pathogenicity of these Amazonian strains.

Concerning the animal strains, our findings reported a heterogeneous virulence score. The lowest value was 2.42 and concerned a strain isolated from a free-living *Panthera onca*, GUY-JAG-2004 [15], while the highest value was 4.90 for the GUY-GAL-VIT0001 strain isolated from a wild animal (greater grison: *Galictis vittata*, Mustelidae) close to an anthropized environment located on the thin coastal border (disturbed forest near the city of Cayenne) [37]. A very high virulence score comparable to that of human strains was calculated for one strain isolated from a dog in the anthropized area (GUY-CAN-FAM0007), whereas those from the other two dogs presented intermediate virulence scores. These two strains which genetically belong to the “Caribbean” 1 and 2 types (respectively GUY-CAN-FAM0001 and GUY-CAN-FAM0002) have been isolated essentially from animals circulating in anthropized areas, and are genotypes that are found throughout the Caribbean and part of South America. They are therefore potentially strains imported by human activity into French Guiana [37]. Little is known about the severity of the symptoms caused by these strains in a contaminated patient. Although an isolated case has demonstrated the opposite, we could expect that they are presumably more symptomatic than those caused by European strains, but still lack the specific severity of Amazonian Toxoplasmosis according to several recorded cases [16, 36].

The lower score of strain GUY-JAG-2004 was interesting because this strain was typed “Amazonian” by Mercier et al. [37]. This strain appeared to have a common ancestor with the type II strain. In a recent classification [33], Guianese strains belong to haplogroups 5 and 10, grouped in clade F and GUY-JAG-2004 is similar with microsatellite typing to a strain isolated from cougars in Canada, belonging to HG11. These strains belong to the ancestral clade D in which there are type II strains. This may explain its lower virulence for mice.

Contrary to our expectations (we had predicted a very high virulence score for the wild strains and a low virulence score for the anthropized strains [36]); there was no obvious correlation between ecological origin and virulence score. Such assays seem interesting to classify strains, rank them by severity, and have a better overview of their potential clinical severity, especially in the case of strains for which little or no clinical information in humans has yet been identified (e.g., Caribbean strains).

Although the number of studied strains was low, the first results revealed that the Guianese strains had a higher virulence than type II strains usually found in Europe and North America. However, virulence is weaker than the highly virulent type I strain. In addition, the study of a larger number of Amazonian strains and reference strains would make it possible to verify our model. Furthermore, the study of strains with a greater number of mice could have an influence on the precision of the virulence score. However, these experiments require the use of a large number of mice, which raises ethical questions.

At present, there is not much information on the virulence mechanisms of these Guianese strains. A study showed the variation of polymorphic rhoptry protein kinases ROP18 and ROP5 of Guianese strains with other strains [39]. Our model would make it possible to develop reliable experimental tests to better describe the genetic or immunological virulence factors of these Amazon strains.

In conclusion, the virulence indicators revealed that the Amazonian strains presented a high virulence profile, but weaker than the highly virulent type I reference. There were differences in virulence between human and animal strains, but also between anthropized and wild strains. In addition to being a clinically relevant animal model of Amazonian Toxoplasmosis, this model could also provide a solid experimental basis for future studies aiming to investigate the underlying pathophysiology of Amazonian Toxoplasmosis.
